# The futuristic manifolds of REM sleep

**DOI:** 10.1111/jsr.14271

**Published:** 2024-06-22

**Authors:** Liborio Parrino, Ivana Rosenzweig

**Affiliations:** ^1^ Sleep Medicine Center University of Parma Parma Italy; ^2^ Neurology Unit Parma University Hospital Parma Italy; ^3^ Sleep and Brain Plasticity Centre, Department of Neuroimaging, Institute of Psychiatry Psychology and Neuroscience (IoPPN), King's College London London UK; ^4^ Sleep Disorders Centre Guy's and St Thomas' NHS Foundation Trust London UK

**Keywords:** Anna Karenina principle, dreams, entropy, gravity, microarousals, predictive processing, rapid eye movement sleep

## Abstract

Since one of its first descriptions 70 years ago, rapid eye movement sleep has continually inspired and excited new generations of sleep researchers. Despite significant advancements in understanding its neurocircuitry, underlying mechanisms and microstates, many questions regarding its function, especially beyond the early neurodevelopment, remain unanswered. This opinion review delves into some of the unresolved issues in rapid eye movement sleep research, highlighting the ongoing need for comprehensive exploration in this fascinating field.

## 1 INTRODUCTION TO THE MANIFOLDS OF RAPID EYE MOVEMENT (REM) SLEEP

On the 4th of September 2023, King's College London virtually hosted a 7‐hr‐long gathering of some of the greatest REM sleep researchers of this and the past century (Parrino & Rosenzweig, 2023); the gathering “The Manifolds of REM Sleep” was otherwise closed to the public. The major impetus was to provide a safe virtual space where debate could be ignited, and experts could share, argue and advance their respective work on REM sleep (Parrino & Rosenzweig, 2023), all whilst marking 70 years since the publication of a pioneering article co‐authored by Eugene Aserinsky and Nathaniel Kleitman, entitled “Regularly Occurring Periods of Eye Motility and Concomitant Phenomena during Sleep” (Aserinsky & Kleitman, 1953).

**FIGURE 1:**
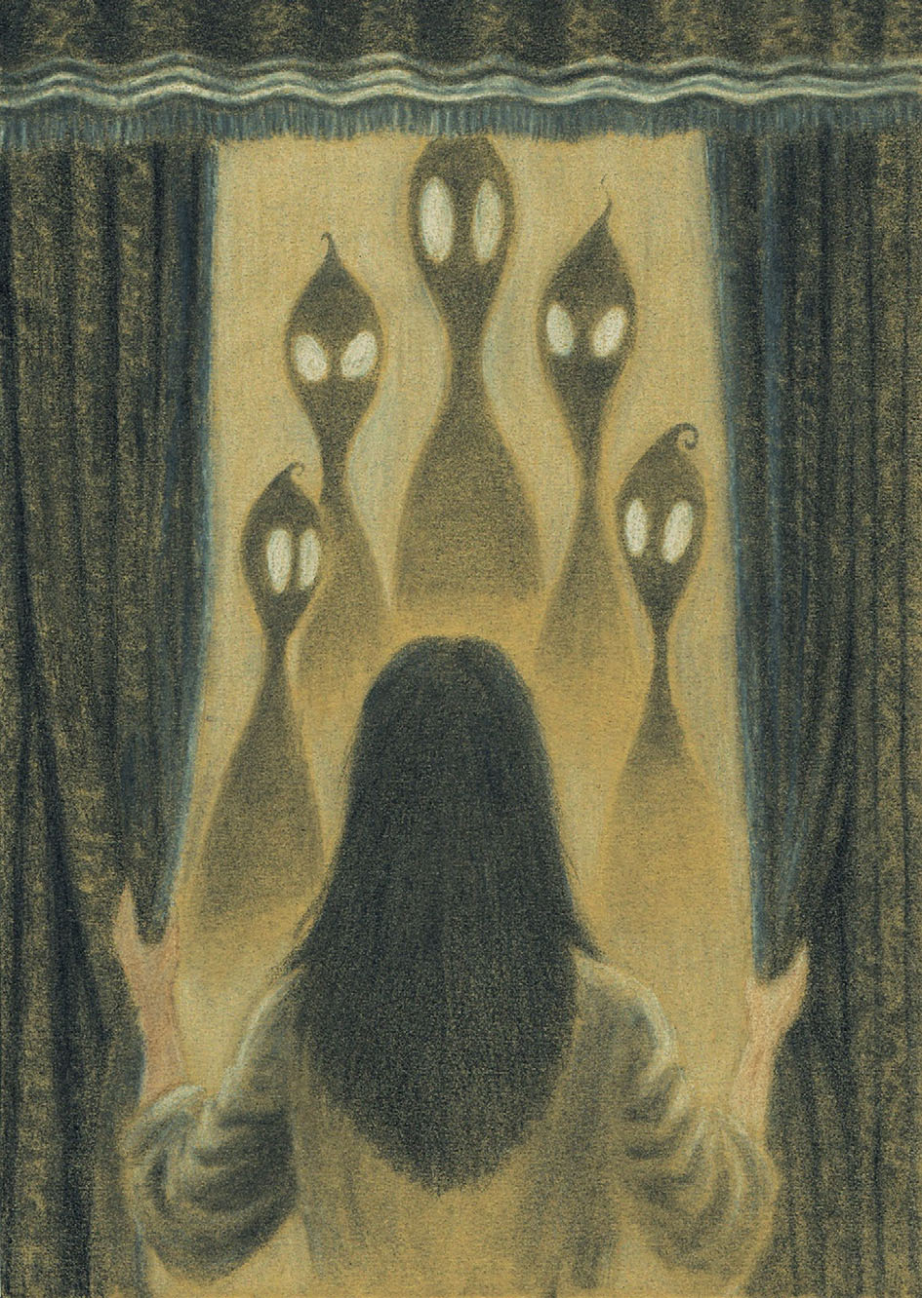
Illustration of a selected oneiric mentation by Davor Aslanovski.

Historically, the oneiric proto‐consciousness of REM sleep has been linked to a primordial state of brain organization (Hobson, 2009). This state, it has been argued, we revert to every night, as to our own adaptive, whilst inactive, theatre of the absurd (Figure 1), in order to reprogramme and “defend” the features specific to our species (Eagleman & Vaughn, 2021; Gonfalone & Jha, 2015; Poole & Rosenzweig, 2020). In keeping with oneiric‐centric view of the REM state, much has been learned over the last 70 years about its role through study of dreams (Schenck, 2025), in heathy and in pathological conditions (Howell & Schenck, 2015; Kang et al., 2023; See et al., 2024; Siclari et al., 2017; Siclari et al., 2020). To date, as also outlined and vehemently argued by the experts in this issue of *Journal of Sleep Research*, the role of REM in neurodevelopment (Blumberg et al., 2022), adult neurogenesis and plasticity (Eagleman & Vaughn, 2021; Kumar et al., 2020), emotional regulation and memory is equally widely recognized (Goldstein & Walker, 2014; Rasch & Born, 2015; Scarpelli et al., 2022; Simor et al., 2020). Nonetheless, the functions and precise mechanisms of this neural state remain obscure (ref Luppi et al., 2024; Simor et al., 2019). Moreover, in line with the paradoxical reputation of REM sleep, more recently *when*, *how* and perhaps, also *who*, should be credited with its discovery, has also been questioned and debated (Denisova, 2024a; Denisova, 2024b).

Despite several fundamental breakthroughs and continued accelerated progress in the field, REM sleep and its unfathomable paradoxical working mechanisms continue to inspire and excite new generations of sleep researchers with its unchartered territories (Parrino & Rosenzweig, 2023). Thus, building on discussions during the gathering for “The Manifolds of REM sleep”, we analyse a major paradigm shift towards a predictive processing approach in REM sleep research, mirroring the one ongoing in cognitive neuroscience (Clark, 2013; Van De Poll & Van Swinderen, 2021).

Predictive processing is a theory in neuroscience, which posits that the brain is constantly generating and updating a mental model of the environment to predict sensory input (Clark, 2013; Friston et al., 2017; Van De Poll & Van Swinderen, 2021). Theoretical ancestors to predictive coding date back as early as 1860 with Helmholtz's concept of unconscious inference (Von Helmholtz & Southall, 1924), whilst more recently a distinctly broad approach to the predictive coding paradigm through the free energy principle has been developed by Friston (2010). This inherently cybernetic approach may prove particularly rewarding for future sleep research, as it assumes that the basic functional architecture of the brain, throughout its whole hierarchy of organization as well as its neural activity across the wake–sleep spectrum, is to compare observations against predictions, and to attempt to operate in such a manner that minimizes prediction errors (Clark, 2013; Friston, 2010; Llewellyn, 2015; Van De Poll & Van Swinderen, 2021).

## 2 ANNA KARENINA PRINCIPLE

A number of pivotal studies over the last few decades have collectively highlighted the critical role of REM sleep in memory replay and consolidation, illustrating the brain's remarkable ability to process, organize and integrate information during sleep (Lewis et al., 2018; Rasch & Born, 2015; Siegel, 2001). Overwhelmingly, REM sleep research over the last few decades has underscored the complexity of sleep‐related memory processes, providing insights into the neural and computational mechanisms that support learning, creativity and memory consolidation (Cai et al., 2009; Llewellyn, 2015).

In parallel, the quest to emulate human intelligence has led to significant advancements in artificial intelligence, particularly through deep learning (Kietzmann et al., 2019; Richards et al., 2019). Deep learning, inspired by the neural networks of the human brain, attempts to replicate its ability to learn, reason and make decisions (Richards et al., 2019). This comparison between deep learning models and the human brain's computing power reveals both the potential and the limitations of current artificial intelligence systems in achieving human‐like intelligence (Kietzmann et al., 2019; Richards et al., 2019). Deep learning utilizes artificial neural networks inspired by the structure and function of the human brain (Kietzmann et al., 2019, Richards et al., 2019). These networks consist of layers of neurons, which process and transmit information (Richards et al., 2019). By adjusting connections and weights based on input data, neural networks can learn and make decisions, paralleling the synaptic plasticity observed in biological brains (Kietzmann et al., 2019, Richards et al., 2019).

Nonetheless, the human brain remains a consummate computational entity, capable of processing complex information with remarkable efficiency and speed due to its intricate network of neurons, enabling it to perform complex tasks, such as emotional reasoning and creative thinking, all of which remain challenging for artificial intelligence (Clark, 2013; Kietzmann et al., 2019; Richards et al., 2019). Comparing deep learning models with the human brain, however, reveals critical insights (Kietzmann et al., 2019, Richards et al., 2019). While deep learning models excel in pattern recognition and data processing, they lack the adaptability, cognitive flexibility and emotional intelligence inherent to the human brain (Richards et al., 2019; Wolpert & Macready, 1997). Furthermore, the brain's ability to learn from minimal data contrasts sharply with the extensive datasets required to train deep learning models, further supporting the argument that the brain may essentially serve as an evolving prediction machine of a never entirely predictable world (Clark, 2013; Friston et al., 2017; Van De Poll & Van Swinderen, 2021). Within this model, periodic neural quiescence and thalamocortical rhythms of non‐REM (NREM) sleep provide essential milieu for the homeostatic synaptic and cell repair mechanisms, growth and development, waste and metabolite clearance, and stress regulation (Tononi & Cirelli, 2020; Van De Poll & Van Swinderen, 2021). On the other hand, a paradoxical neural activity of REM sleep may be seen as a fundamental evolutionary strategy for simulation and testing of a broad range of internal models, during limited exposure to the environment (i.e. sleep), against the ever‐changing, highly unpredictable, wakefulness landscapes where unexpected events typically evoke prediction error signatures (Clark, 2013; Llewellyn, 2015; Simor et al., 2022; Van De Poll & Van Swinderen, 2021).

Arguably, in human brains, any mismatched expectations become associated with an emotional response (Van De Poll & Van Swinderen, 2021), with significant, strengthening implications for our memory, and memory consolidation of these events (Goldstein & Walker, 2014; Llewellyn, 2015). Within this construct, as elegantly proposed by Van de Poll and Van Swinderen (2021), emotional responses and their positive or negative “valence” signals may simply represent an ancient evolutionary mechanism designed to potently refine and generalize internal models of the world, and minimize prediction errors (Clark, 2013; Van De Poll & Van Swinderen, 2021).

Paradoxically, however, it might not be in our “evolutionary” interest to entirely eliminate error detection and surprise, which can occur by generalization or habituation processes (Van De Poll & Van Swinderen, 2021).This philosophical, logical and mathematical conundrum has been a matter of debate through centuries (Aristotle, Ross, & Brown, 2009; Clark, 2013; Friston et al., 2017; Gill, 2021). Depending on the scientific field, it sports disparate names, including that of Tolstoy's Anna Karenina principle (Gill, 2021). The Anna Karenina principle is named after the opening sentence in the eponymous novel: *Happy families are all alike; every unhappy family is unhappy in its own way* (Gill, 2021; Tolstoy, 1889), and it suggests that successful entities share common traits, whilst failure can occur due to deficiencies in any of these traits (Gill, 2021). This principle has intriguing implications when applied to the field of neuroscience, particularly in understanding the brain's predictive processing, as it implies that successful cognitive processing relies on a set of common factors: accurate sensory data, efficient neural computation, and effective updating of predictive models during REM sleep (Simor et al., 2022). Failure in any one of these areas can lead to disorders of interoception, emotional dysregulation and cognitive errors, such as misperceptions, hallucinations or illogical reasoning (Clark, 2013; Llewellyn, 2015). For instance, accurate sensory data are crucial for effective predictive processing. When sensory inputs are compromised, as in the case of extreme environments, sensory deprivation or distortion, the brain's predictive models can become inaccurate, leading to perceptual anomalies (Llewellyn, 2015). Similarly, efficient neural computation is necessary for the timely and accurate updating of predictive models (Clark, 2013; Van De Poll & Van Swinderen, 2021). Delays or inaccuracies in neural computation can impair cognitive functions, leading to slow or incorrect responses to environmental changes (Simor et al., 2022). Finally, the brain must effectively update its predictive models based on new sensory information through effective and efficient sleep cycling during the night (Simor et al., 2022). Failures in this process can result in the persistence of outdated or incorrect beliefs, impacting decision‐making and behaviour (Llewellyn, 2015).

Therefore, REM‐associated neural activity may effectively serve to quantify the brain's entropy (i.e. a quantity used for measuring uncertainty about the state of a system; Friston et al., 2017), and perhaps also to ensure that we remain safely in our own distinct form of existential “unhappiness”, as the pure happiness, in an ever‐changing alien world, might be too costly, and metabolically and neurocomputationally unreachable (Hobson et al., 2021). Excitingly, this principle may also, at least in part, explain the reported evolutionary negativity bias of the human brains, as well as the potential role of REM sleep in its regulation (Goldstein & Walker, 2014; Lazarus, 2021).

Thus, hypothetically speaking, the Anna Karenina principle applied to the field of REM sleep function may underscore the complexity of cognitive processing, REM sleep neural activity, and the multitude of factors that must align for accurate perception and reasoning, and why it might not be in our evolutionary interest to “code” for the “perfect predictions” (Clark, 2013, Llewellyn, 2015). It could be also argued that it may similarly highlight the importance of a holistic approach to studying the brain, considering not just the individual components of predictive processing, but also the role for sleep physiology and the intricate interplay between them (Clark, 2013).

Intriguingly, given the strong links of REM sleep with emotional regulation, the dysregulation of which constitutes an elementary fabric of all cognitive disorders, and it is a symptom of most, if not all, neuropsychiatric and sleep disorders (Goldstein & Walker, 2014; Rosenzweig et al., 2015; Rumble et al., 2015), this view has some interesting inferences for memory, awareness, notably, also for future translational clinical implications (for more in depth coverage of these fields, please refer to Hobson et al., 2021; Rasch & Born, 2015).

## 3 DISTINCT REM SLEEP STATES ON A WAKE–NREM–REM SLEEP CONTINUUM

Traditionally, it has been argued that REM sleep encompasses several paradoxical brain states of intense cortical activity and muscle atonia (Simor et al., 2019; Simor et al., 2020; Simor et al., 2021). Of these, the most easily recognizable are the phasic and tonic REM, which significantly differ in awakening thresholds, sensory processing (Wei & Van Someren, 2020), cortical oscillations (Simor et al., 2019), the molecular and cellular machinery (Yamada & Ueda, 2019), and notably in the activated neurocircuitry (Luppi et al., 2024).

Recently, this historic conceptualization of NREM, REM and wake continuum as mutually exclusive global brain states with contrasting polygraphic signatures (Simor et al., 2020) has been challenged by a body of work on the brain's phasic microarousals or “avalanches”, which have been reported to occur regardless of the brain's NREM, REM continuum (Ramirez‐Villegas et al., 2021; Scarpetta et al., 2023; Simor et al., 2020; Tsunematsu et al., 2020). For example, it has been recently shown that the alternating spatial–temporal coupling between phasic ponto‐geniculate‐occipital‐waves and hippocampal sharp wave ripples and hippocampal theta, previously classically associated with distinct REM or NREM sleep stages, may promote systems and synaptic memory consolidation, as well as synaptic homeostasis (Tononi & Cirelli, 2014; Tononi & Cirelli, 2020; Tsunematsu et al., 2020).

Of note is that it has been argued that similar mechanisms may also underlie the abstraction or generalization or forgetting processes that occur during sequential activation and reorganization of human hippocampal “concept cells” (Quian Quiroga, 2020). Concept cells have been characterized as neurons of relatively late latency of responses, described in the human medial temporal lobe, selectively responding to distinct personally relevant concepts with a high degree of multimodal invariance, irrespective of the context (Quian Quiroga, 2019; Quian Quiroga, 2023). This is important as these cells are yet to be demonstrated in other animal models (Quian Quiroga, 2023). Thus, the very concept that human episodic memories can be coded by hippocampal context independent and invariant engrams suggests crucially different neuronal coding principles may be at play during human sleep (Quian Quiroga, 2023). Perhaps in further agreement with this, a recent finding suggests that human perception, whilst experienced as a continuous flow, counterintuitively seems to rely on a discrete sampling mechanism during distinct windows of permissiveness (Michel et al., 2020). Indeed, this may suggest that human episodic memory, unlike that of other species, may be more like semantic memory (Quian Quiroga, 2019) and less as a hippocampal embedded mental time travel (Quian Quiroga, 2020). If proven, this may have fundamental implications for future studies of the role of REM sleep in memory processing (Ben Simon et al., 2022).

Moreover, in this background, the proposition from Van De Poll and Van Swinderen (2021) that REM sleep should be re‐examined as a conserved sleep function that co‐evolved alongside selective attention to maintain an adaptive balance between prediction and surprise undertakes a special meaning. Intriguingly, this idea is not new, and the suggestion that sleep utilizes some of the attentional/arousal neurocircuitry has been advanced in earlier work by several groups that linked the brain's arousal system and microstructure of sleep (Gnoni et al., 2021; Halasz, 1998; Halasz et al., 2004; Simor et al., 2022; Van De Poll & Van Swinderen, 2021).

## 4 SHARED ARCHITECTURE: AROUSAL AND REM SLEEP

According to the American Academy of Sleep Medicine (AASM) scoring rules, an electroencephalogram (EEG) arousal is an abrupt shift in EEG frequency including alpha, theta and/or frequencies greater than 16 Hz (but excluding the spindle band), which lasts for 3–15 s (Berry et al., 2012). When they also occur during REM phase, the EEG features must be accompanied by an increase in electromyography (EMG) signal of at least 1 s (Berry et al., 2012). Whilst it is widely accepted that the standardized criteria are mandatory to avoid Babel–Tower phenomena among the experts of a common clinical or scientific field, including in sleep medicine, to date the recommended arousal scoring thresholds remain prodigiously arbitrary, with limited or no evidence, and at best confusing despite the very best efforts to address this issue (Berry et al., 2012). For instance, a concomitant muscle activation is cited as necessary in order to recognize an arousal in REM sleep, a condition otherwise dominated by a complete EMG atonia. This (erroneously) suggests that no cognitive, autonomic or neurophysiological arousal may occur in the absence of a simultaneous increase of muscle tone. Moreover, and perhaps even more puzzlingly, the scoring requirements similarly include a precise 1‐s length of EMG reinforcement, bar clear evidence for this time period being of specific importance.

Clinically, the identification of EEG arousals in REM sleep has highlighted the existence of a distinct condition, the REM sleep instability (Feige et al., 2023). The REM sleep instability has recently been posited as an important pathomechanistic entity, especially in patients with insomnia (Feige et al., 2023). Nonetheless, the quantitative information on the definition of what exactly is a state of REM instability is still lacking. For example, how many EEG arousals per minute are required before we can define it as such, also what duration of wakefulness intrusions should count, and what should the interval between successive EEG arousals be in order for us to consider the two events functionally connected or separated? These are all pertinent, however, as of yet undefined parameters that may mask any potentially adaptive role for these intrusions (Selbaek‐Tungevag et al., 2023; Wong & Lovier, 2023), and in the absence of which, the term REM instability remains a fascinating though complex issue (Parrino & Vaudano, 2018).

In keeping with this, a body of work suggests that across the conscious sleep–wake continuum, circadian and homeostatic processes generate distinct phasic (micro) arousals, such as ultradian cyclic alternating patterns (Halasz et al., 2004). Whilst the brain's exact circuitry and mechanistic platform of these remains elusive, it has been advanced that they may rhythmically arise due to the interplay of the brainstem's autonomic, arousing and respiratory pacemakers (e.g. parabrachial, Kölliker–Fuse, locus coeruleus complex; Yang et al., 2021), respiratory preBötzinger Complex (Anaclet et al., 2014; Datta, 2006; Ramirez‐Villegas et al., 2021; Tsunematsu et al., 2020; Xu et al., 2021). Importantly, these microarousals appear to gate and initiate “avalanches” across the NREM–REM–wake continuum (for a more in depth discussion, please also see Halasz et al., 2004; Parrino & Vaudano, 2018; Scarpetta et al., 2023). It has been argued that it is this precise interplay that ensures sleep's instability, as well as its resilience (Parrino & Vaudano, 2018). Moreover, these events further enable the localized phasic‐events‐rich windows (Buzsaki, 2015), thus facilitating integration of the information (Tononi, 2004) about our own previous states, as well as enabling incorporation of the incoming ascending sensory volleys across different behavioural brain states (Scarpetta et al., 2023). Moreover, by acting as gating arousing mechanisms towards state‐dependent global coordination (Ramirez‐Villegas et al., 2021; Tsunematsu et al., 2020), phasic microarousals may not just contribute to the reorganization of cortical/subcortical networks during sleep (Cirelli & Tononi, 2021; Nitzan et al., 2020; Parrino & Vaudano, 2018; Tononi & Cirelli, 2014; Tononi & Cirelli, 2020; Tsunematsu et al., 2020), but they may provide the very skeleton of our consciousness (Tononi, 2004). In line with this idea, the most state of the art mathematical models of sleep suggest that arousal state feedback, to either homeostatic or circadian drive, may indeed act as a potent physiological generator of an ultradian 90–120‐min (wake) REM–NREM continuum (Park et al., 2021; Phillips et al., 2013).

More recently, generation of microarousals and phasic events has been shown to also occur in a periodic pattern during NREM sleep in rodents, riding on the peak of locus coeruleus‐generated infraslow oscillations of extracellular noradrenaline (Kjaerby et al., 2022). Conversely, during the descending phases of noradrenaline oscillations, a distinct drive towards sleep spindle and REM sleep generation was demonstrated (Kjaerby et al., 2022; Logothetis et al., 2012; Muehlroth et al., 2019; Osorio‐Forero et al., 2021). Arguably, if this process is also proven in humans, this may suggest that manipulation of noradrenergic oscillatory amplitude could be an august transdiagnostic target for improving REM sleep and cognition (Kjaerby et al., 2022).

## 5 FUTURISTIC INTER‐STELLAR MANIFOLDS OF REM SLEEP

Alas, habituating geopolitical and scientific landscapes of the 21st century maintains some absurd and overly complex historical patterns of the early 20th century. Can we as scientists help make sense of the world around us, and perhaps advance some of that knowledge to avoid mistakes of the past?

Fast‐forwarding to our times of restricted and unsustainable resources, ageing population, increased global migration and endangered habitats, it may be important to consider which new, pioneering sleep research trajectories may accrue the most beneficial returns (Buguet, 2007; Minor et al., 2022). Arguably, climate change, rising global, and in particular nocturnal, ambient temperatures, along with increased prevalence of insufficient sleep worldwide have all been projected to contribute to worsening of societal inequalities (Minor et al., 2022).

In this context, it is important to note that REM sleep may be particularly vulnerable to extremes in environmental temperatures (Buguet, 2007), with women more effected than men under identical conditions (Minor et al., 2022). It has been argued that short of global political action that would lead to drastic greenhouse gas concentrations stabilization by the end of the century, each of us may be subjected to an additional 2 weeks of temperature‐attributed short sleep each year (Minor et al., 2022). In the harsher world of our future, this may matter, as some of the recent research points to a strong link between shorter sleep, especially REM sleep, and an overall decreased altruism observable at three different societal scales: within individuals, across individuals and at a nationwide level (Ben Simon et al., 2022; Gnoni et al., 2023).

Lastly, as humanity stands on the cusp of interstellar travel, understanding and managing human physiology, particularly sleep, is becoming paramount (Pavy‐Le Traon & Taillard, 2010). Only recently, several international space agencies have jointly announced that the first humans might be sent on a 7‐month‐long journey to Mars as early as the 2030s, and that this journey will involve the development and habitation of a lunar station (Jones et al., 2022; Luo et al., 2022). Within this context, it is important to recall sleep's major hypothesized role in regulating the complex organization of brain dynamics by keeping excitatory and inhibitory processes balanced (Parrino & Vaudano, 2018). While prolonged wakefulness increases brain excitability, sleep reduces it, preventing an imbalance towards excitation that would favour uncontrolled runaway activity (Parrino & Vaudano, 2018; Priesemann et al., 2013). Similarly, whilst the mechanics of circadian regulation of the brain's resilience remain elusive, the emerging picture is one of circadian rhythms' anticipation, and the regulation of the prefrontal cortical (messenger RNA) transcriptome, which is then translated during the sleep–wake cycles (Noya et al., 2019). Arguably, an abnormal circadian phase and aberrant (REM) sleep physiology will impact on the homeostatic regulation of cortical arousal, and likely lead to abnormal vigilance, emotional and cognitive dysregulation, including increased susceptibility to distinct neuropsychiatric disorders (Hupfeld et al., 2021; McCarthy et al., 2022).

Moreover, microgravity itself significantly alters sleep quality and duration, as evidenced by numerous spaceflight studies (Hupfeld et al., 2021; Rosenberg & Angelaki, 2014). Astronauts often experience shorter sleep periods, increased sleep fragmentation and a reduction in slow‐wave sleep (Stickgold et al., 2020). These changes are attributed to factors such as the absence of gravity‐dependent sleep cues, altered light exposure, and the psychological stress of confinement and isolation (Jones et al., 2022). The implications of these disruptions extend beyond mere fatigue, affecting cognitive performance, mood and critical mission operations (Hupfeld et al., 2021).

Historically, the first sleep EEG in space was conducted during the 14‐day Gemini VII mission in December 1965 (Maulsby, 1966). Since then, a body of work by the international space agencies has shown marked impact on sleep efficiency, duration, as well as shifts in sleep architecture compared with baseline on the Earth, which can evolve over the course of the mission (Stickgold et al., 2020; Stoilova, Zdravev Yanev and Zdraveva, 2000; Stoilova, Zdravev and Yanev, 2000; Stoilova, Zdravev and Yanev, 2003). On average, in space, time in REM sleep is decreased, and REM presents with aberrant microstructure (Jones et al., 2022; Stickgold et al., 2020). Of note is that the distinct phenotype of REM sleep under conditions of microgravity has previously also been demonstrated by Gonfalone and colleagues (Gonfalone, 2019). Gonfalone's work in the Microgravity Department at the European Space Agency where he meticulously collected the European Astronaut Corps' reflections on the influence of gravity on sleep and wakeful functioning (Gonfalone, 2019) also led to his later extrapolation for the role of gravity on REM sleep and an overall sleep architecture in other animal species (Gonfalone, 2019).

Gravity is a defining force that anchors our perception of the environment, and that enforces fundamental constraints on our interactions with the world (Dakin & Rosenberg, 2018). On this planet, our vertical posture confers numerous benefits for us humans, but it also renders us less stable and more susceptible to falls (Dakin & Rosenberg, 2018). Maintaining verticality requires that we estimate our orientation relative to gravity from noisy and ambiguous sensory signals (Dakin & Rosenberg, 2018; Rosenberg & Angelaki, 2014). Moreover, sensory systems encode the environment in egocentric reference frames, creating inherently unstable representations that shift and rotate as we move (Rosenberg & Angelaki, 2014). Where and how the brain transforms these signals into an allocentric, gravity‐centred representation of the world that is stable and independent of the observer's spatial pose remains unknown (Rosenberg & Angelaki, 2014). In this respect, Gonfalone and colleagues argued a critical role for REM sleep (Gonfalone, 2019; Gonfalone & Jha, 2015). For instance, they advanced that REM‐associated atonia in terrestrial animals may specifically affect antigravity muscles ensuring reduced vigilance against a fall during sleep, thus ensuring a smooth transformation from ego to allocentric dreamscapes or REM sleep (Gonfalone, 2019, Gonfalone & Jha, 2015). Accordingly, recent studies of re‐enacted behaviours of dreams occurring in patients with REM behaviour disorder, where there is an inherent loss of this atonia, suggested predominantly allocentric spatial navigation during mentations of REM sleep (See et al., 2024; Wasserman et al., 2022).

In conclusion, addressing extreme environmental conditions, microgravity states, and their overall impact on the sleep and circadian physiology will be crucial for the development of effective countermeasures and technologies to support extended space missions of the 21st century (Luo et al., 2022; Pavy‐Le Traon & Taillard, 2010).

## 6 CONCLUSION

In summary, while significant strides have been made in understanding the intricacies of REM sleep and its pivotal role in cognitive functions, emotional regulation and overall health, substantial gaps in our knowledge persist. These gaps not only limit our comprehension of the underlying mechanisms of REM sleep, but also impede the development of targeted interventions for sleep disorders and related cognitive and emotional disturbances. As discussed during the gathering “The Manifolds of REM Sleep” (Parrino & Rosenzweig, 2023), addressing these unmet needs requires a multidisciplinary approach that spans computational neuroscience, molecular biology, neurophysiology, cognitive neuroscience and clinical research (Llewellyn, 2015). By charting a course through the unexplored territories of REM sleep, future research has the potential to unravel the complexities of this unique state of consciousness, paving the way for innovative treatments that enhance cognitive health and well‐being, and that expand our understanding of the human mind.

## AUTHOR CONTRIBUTIONS

**Liborio Parrino:** Conceptualization; writing – original draft. **Ivana Rosenzweig:** Conceptualization; writing – original draft.

## CONFLICT OF INTEREST STATEMENT

The authors declare no conflict of interest.

## Data Availability

Data sharing not applicable to this article as no datasets were generated or analysed during the current study.
